# The effect of dragon-kings on the estimation of scaling law parameters

**DOI:** 10.1038/s41598-020-77232-6

**Published:** 2020-11-19

**Authors:** Carmen Cabrera-Arnau, Steven R. Bishop

**Affiliations:** Department of Mathematics, University College London, Gower Street, London, WC1E 6BT UK

**Keywords:** Applied mathematics, Scientific data, Statistics

## Abstract

Scaling laws are used to model how different quantifiable properties of cities, such as the number of road traffic accidents or average house prices, vary as a function of city population size, with parameters estimated from data. Arcaute et al. raised the issue of whether specific cities with extremely large population sizes, known as dragon-kings, should be considered separately from other smaller cities when estimating the scaling law parameters since the two types of cities tend to display different behaviour. Through the analysis of randomly generated samples, we find that the inclusion of dragon-kings in the scaling analysis does not affect the estimated values for the parameters but only provided that all the data points satisfy the same scaling law. We also analyse randomly generated samples where data corresponding to a particular city deviates from the scaling law followed by the rest of the cities. We then show that deviations corresponding to dragon-king cities have the most significant effect on the estimated values of the scaling parameters. The extent of this effect also depends on which estimation procedure is used. Our results have important implications on the suitability of scaling laws as a model for urban systems.

## Introduction

Due to the shift towards a mostly urbanised world^1^, there is now an increasing interest in discerning the relationship between the population size of a city and its environmental or socioeconomic characteristics. This relationship is often mathematically modelled as a scaling law, whereby a quantifiable property *Y* is postulated to vary with city population size *X* according to

1 $$\begin{aligned} Y(X) = \alpha X^{\beta }. \end{aligned}$$

In Eq. (1), $\alpha$ and $\beta$ are known as scaling parameters: parameter $\alpha$ is a proportionality constant and parameter $\beta$ is the scaling exponent. According to the value of the scaling exponent, the scaling law can display three types of behaviour. If $0<\beta <1$, the scaling law has sublinear behaviour, which implies that larger cities register lower values of *Y* per capita. If $\beta = 1$, the scaling law has linear behaviour and the values of *Y* per capita are constant across population sizes. If $\beta > 1$, *Y* is said to increase superlinearly with city population size. When *Y* increases superlinearly, the values of *Y* per capita are higher for more populous cities. It has been found that urban properties such as the average water consumption^2^ grow linearly with city population size; economic productivity^3^ or the amount of coverage received by the media^4^ grow superlinearly and the number of road traffic accidents in cities grows sublinearly^5^.

However, it is challenging to quantify reliably from data the value of the scaling exponents and their estimates usually come with caveats^6–9^. For example, Arcaute et al.^10^ raise the issue that, on the one hand, cities with extremely large populations such as London in the UK, tend to behave differently from the rest of cities within the urban system due to their socioeconomic role; therefore, they should possibly be analysed relative to other cities with extremely large populations and separately to the rest of cities within an urban system. On the other hand, these extremely populous cities act as hubs, making the urban system highly integrated. From this point of view, all the cities in the urban system should be analysed together despite the fact that the value of the estimated scaling parameters might be dominated by the unusual behaviour of extremely populous cities.

Rather than considering extremely populous cities as a separate category of cities, in this paper we aim to understand how their inclusion might affect the estimated values of the scaling parameters. This approach is philosophically in line with the complex systems perspective by which systems formed by many interacting parts should be considered as a whole to properly understand their behaviour. In order to specify what we mean by extremely populous cities, we use the statistical concept of dragon-king^11^. A ‘dragon-king’ is defined as an event that, due to its large size, is statistically and mechanistically considered to be an outlier of the underlying heavy-tailed probability distribution followed by the rest of events. Sornette shows that dragon-kings can be present in the distribution of city population sizes which, as is the case for many other systems in nature, follows a heavy-tailed probability distribution.

The introduction of dragon-kings motivates the statistical approach that we take here. This is manifested by the fact that we model *X* as a random variable which follows a heavy-tailed probability distribution. Variable *Y*, which could for instance represent the number of road accidents in a city, is also modelled as a random variable. The scaling law in Eq. (1) hypothesises that *Y* is related to variable *X* and consequently, *Y*’s probability distribution must be conditional on *X*. Therefore, as Leitão et al.^12^, we interpret the expression given in Eq. (1) as

2 $$\begin{aligned} \text{ E }[Y|X] = \alpha X ^{\beta }. \end{aligned}$$

The presence of dragon-kings in data related to variable *X* can be detected through the application of statistical tests. For example, Pisarenko and Sornette^13^ introduce two tests that work when *X* is assumed to follow a particular type of heavy-tailed distribution: the power-law probability distribution. Similarly, Janczura and Weron^14^ propose a more generalised test, where *X* can be compatible with any heavy-tailed probability distribution. However, in all these instances, the underlying distribution of *X* has to be known. Although further statistical tests such as that proposed by Clauset et al.^15^ can help decide whether a data sample is compatible with a given hypothesised distribution, they cannot guarantee whether the data is actually drawn from that distribution. Identifying the true underlying probability distribution of *X* is therefore challenging and it is particularly so when the distribution is believed to have a heavy tail. This is partly due to the fact that the number of data points in the upper tail of empirical heavy-tailed distributions is often scarce. These complications are captured in the disagreements between Eeckhout, Levy and Malevergne et. al concerning the probability distribution of city population sizes^16–19^.

We consider here that the use of synthetic data is more appropriate for a discussion in the methodology, given the difficulty in determining the distribution of empirical data regarding city population sizes. Firstly, we generate samples for variable *X* with an underlying heavy-tailed distribution whose parameters are known. We then use statistical tests to classify these samples according to whether or not they contain a dragon-king. We generate associated random data for *Y* so that it satisfies some scaling law. The use of synthetic data allows us not only to detect dragon-kings unequivocally via the application of statistical tests, but also to know the true values of the scaling parameters $\alpha$ and $\beta$. We can then use different estimation methods to obtain the values of the scaling parameters from the randomly generated samples and compare these with the true values. Discrepancies between the estimated scaling parameters and the true parameters can then be assessed.

The contribution of our work is mainly methodological: we demonstrate that different estimation methods account for dragon-kings in different ways and under certain circumstances, this can result in different estimated values for the scaling parameters. Our contribution is also somewhat fundamental since our findings suggest that there might be inherent limitations in the precise estimation of scaling parameters from real world data.

The structure of the paper is as follows. In “Methods” section, we give precise details of the algorithm to generate samples as well as the methods for the estimation of the scaling parameters. “Results” section is devoted to explaining the results of our analysis, where samples without dragon-kings are used as a null model and are then compared to samples with one dragon-king. Additionally, samples where a city (the dragon-king in particular) follows a different scaling law than the rest of the cities in the sample are also analysed. In “Discussion” we discuss the implications of our results on the precise estimation of scaling parameters from real world data.

## Methods

### Generation of random data samples

In essence, the approach that we take here involves assessing the discrepancies between the values of the scaling exponents estimated from synthetic data and their known true values. We start by producing samples of $n=500$ simulated cities, where each city has an associated value of *X* and *Y*. The population sizes of these cities are randomly generated so that the underlying probability distribution followed by *X* is a power-law with exponent $\gamma$. Hence, the random variable *X* takes the value *x* with probability given by

3 $$\begin{aligned} \text{ Pr }(X=x) = \dfrac{\gamma - 1}{x_{min}}\Big (\dfrac{x}{x_{min}}\Big )^{-\gamma } \end{aligned}$$

for $x \ge x_{min}$. Even though the power-law distribution is defined for continuous random variables, it can still give a good approximation of the distribution of city population sizes provided that $x_{min}$ is sufficiently large^15^. This particular choice of heavy-tailed distribution allows us to later apply the statistical tests proposed by Pisarenko and Sornette^13^ for detection of dragon-kings in the samples.

A variety of probabilistic models can be used to generate the value of *Y* associated to each city. In this paper, two cases are explored that typically arise when analysing real data. For the first case, *Y* is assumed to have a Poisson probability distribution conditional on the value *x* of variable *X*. The probability that *Y* takes the value *y* is given by

4 $$\begin{aligned} \text{ Pr }(Y=y|X=x) = \dfrac{\mu ^{y} e^{-\mu }}{y!}, \end{aligned}$$

where parameter $\mu$ is equal to the expected value of the distribution $\mu = E[Y|X] = \alpha X^{\beta }$ and the variance is also equal to the expected value. A Poisson distribution is frequently used to model the probability of the number of occurrences of particular events in a fixed time or space interval, if these events occur with a known constant mean rate. However, as a consequence of heterogeneity in real data (e.g. data collected on different weekdays, times of the day, etc.) and the omission of relevant explanatory variables, real data sets tend to display overdispersion, that is, a variance larger than the mean^20^. In order to account for this overdispersion, a second probability model for the generation of *Y* is explored here. In this case, *Y* is modelled through a negative binomial (NB) probability distribution, also conditional on *X*, which can be parametrised in terms of the mean $\mu$ and a parameter *r* that is related to the degree of overdispersion. Then, *Y* takes the value *y* with the following probability conditional on $X=x$

5 $$\begin{aligned} \text{ Pr }(Y=y|X=x) = \left( {\begin{array}{c}y + r-1\\ y\end{array}}\right) \Big (\dfrac{\mu }{r + \mu }\Big )^r\Big (\dfrac{r}{r+\mu }\Big )^k. \end{aligned}$$

The mean of the distribution is again $\mu = E[Y|X] = \alpha X ^{\beta }$, however, the variance $\sigma ^2$ now varies with $\mu$ according to $\sigma ^2 = \mu + \frac{1}{r}\mu ^2$.

Below, we give the specific algorithm that we use to generate samples where all the data points obey Eq. (2):

1. We start with $m=0$ samples without dragon-kings and $m=0$ samples with one dragon-king.
2. If $m = 1000$ the algorithm is completed; otherwise, we follow the steps below:
  - We draw a random sample of size $n=500$ for variable *X* from a power-law probability distribution with parameters $\gamma = 2.2$ and $x_{min} = 15000$. We denote the *n* values by $x_i$, with $i = 1,...,n$ so that $x_1 \ge ... \ge x_n$.
  - We transform the sample by taking $x_1$ and multiplying it by 100. We denote the new $x_1$ by $x_{DK}$.
  - We check whether the original and the transformed random samples contain dragon-kings. If there is one dragon-king in the transformed sample and zero dragon-kings in its original form, we proceed to the next steps. Otherwise, we discard the original and transformed samples and go back to step 2.
  - For each value $x_i$ in the original sample, we draw an associated value $y_i$ from a probability distribution that satisfies $E[Y=y_i|X=x_i] = \alpha x_i ^{\beta }$. The true values of the scaling parameters are set to $\alpha = 0.01$ and $\beta = 1.15$. For the transformed sample, the values of *Y* are kept the same for $i = 2, ..., n$ but $y_{DK}$ is now conditional on $x_{DK}$.
  - We store the values of *X* and *Y* corresponding to the original and transformed samples and increase *m* by one.
  - We return to step 2.

We use the package ‘powerlaw’^21^ to generate the power-law distributed values of *X* in step 2.(a). To check for the presence of dragon-kings in step 2.(c), we apply the DK-test and the U-test proposed by Pisarenko and Sornette^13^. For the U-test, instead of finding the parameter values through the maximum likelihood method as prescribed by the authors, we simply set them to the true values, i.e. those used in step 2.(a) to generate the data related to *X*. We consider that a sample does not contain any dragon-kings if (i) the *p*-values given by the U-test are above 0.1 for the top 100 cities, ranked by decreasing population size and (ii) the *p*-values given by the DK-test are all above 0.1 when we compare the first spacing to a number of spacings ranging from 1 to 25. We consider that a sample transformed according to step 2.(b) has one dragon-king if (i) the *p*-values given by the U-test corresponding to the top 100 cities, ranked by population size, are above 0.1 except for the largest city, which must be below 0.1 and (ii) the *p*-values given by the *DK*-test are all below 0.1 when we compare the first spacing to a number of spacings ranging from 5 to 25.

As established by Sornette and Ouillon^22^, dragon-kings are extreme events that arise from generating mechanism which are not necessarily active for the rest of the entities under consideration. As a consequence, dragon-kings tend to display unique behaviours and in the context of scaling laws, this may have an impact on the estimated values for the parameters. Indeed, Gomez-Liévano et al.^23^ show that the values of *Y* associated with extremely populous cities are prone to very large variance and this can result on biased estimators for the scaling exponents. To test these effects, here we also generate samples where the value of *Y* associated with $x_{DK}$, denoted by $y_{DK}$, deviates from the scaling law satisfied by rest of cities and instead, behaves according to a scaling law with a different exponent. We generate this type of samples by using the same algorithm as before but in step 2.(d) $y_{DK}$ is generated ensuring that $E[Y|X] = \alpha X^{\beta ^*}$, where $\beta ^* = 0.5$. The effect of these deviations associated with dragon-kings on the estimation of scaling exponents can be compared with the effect of deviations associated with other cities. In order to do this, we generate additional samples where the value of *Y* corresponding to a different city of smaller population size is generated according to $E[Y|X] = \alpha X^{\beta ^*}$, while for all the other cities $E[Y|X] = \alpha X^{\beta }$ is satisfied.

### Estimation of scaling parameters $\alpha$ and $\beta$

We generate the values of *Y* associated to the cities in each sample following a known distribution conditional on *X*. When *Y* follows a Poisson distribution, the Poisson regression must be applied to estimate the scaling parameters. Similarly, when *Y* has a NB distribution, the negative binomial regression must be applied. However, it can be difficult to ascertain the distribution of *Y* when real data is considered and this can lead to the wrong choice of regression model. Hence, in order to show how this can affect the estimated values for the scaling parameters, in “Results” section we also apply the Poisson regression to samples where *Y* is NB-distributed and the negative binomial regression to samples where *Y* is Poisson-distributed.

Different sets of $m=1000$ samples are considered in “Results” section. For each of these sets, we estimate the scaling parameters via one of the above mentioned regression methods (a total of 1000 regressions for each set). This allows us to obtain a distribution for the estimated parameters and hence, provide 90% confidence intervals based on the 5th and 95th percentiles.

## Results

### Samples where all values of *Y* satisfy the same scaling law

**Figure 1:**
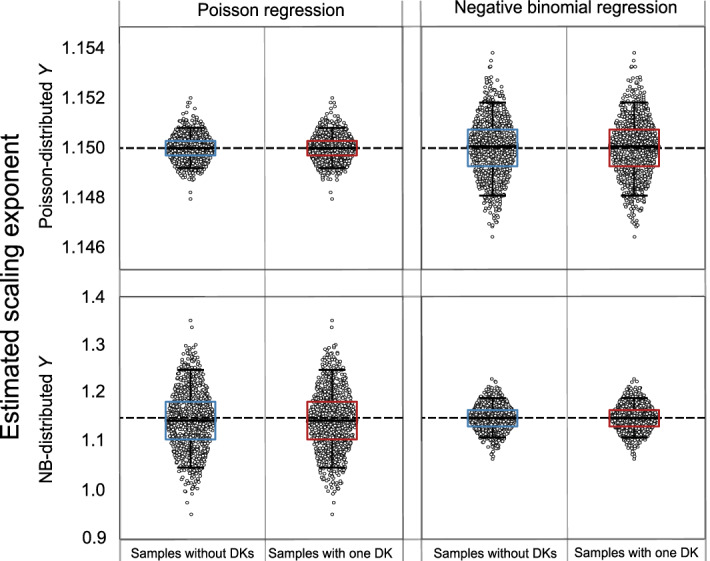
Scatter plots and boxplots showing the distribution of estimated values for parameter $\beta$ from each of the $m=1000$ samples with $n=500$. The bottom and top of the box indicate the first and third quartiles, the middle line in the box is the median and the upper and lower limits correspond to the 5th and 95th percentiles, giving a 90% confidence interval. The horizontal dashed line represents the true value of $\beta$, used to generate the synthetic data samples.

Figure 1 displays the estimated values of the scaling exponent obtained from performing simulations on different sets of randomly generated samples. Only the estimated exponent is shown here because it generally is the parameter of interest. The samples used to produce the left-most scatter plots and boxplots in each of the four panels do not contain any dragon-kings, whereas those used to obtain the right-most scatter plots and boxplots have exactly one dragon-king. For each sample, we generated the *Y*-data so that it satisfies Eq. (2) with $\beta =1.15$. We observe that for each probabilistic model and regression method, the distribution of estimated values for parameter $\beta$ remains pretty much the same for samples with and without dragon-kings. This suggests that having a dragon-king does not change the results of the scaling analysis as long as $E[Y|X] = \alpha X ^{\beta }$ holds for all cities.

The results corresponding to the top-right and bottom-left panels in Fig. 1 show the effects of applying a regression method that assumes a probability model for *Y* other than the actual distribution followed by the *Y*-data. For example, if a negative binomial regression is applied to samples with Poisson-distributed *Y*, this results in a distribution for the estimated values of $\beta$ which is a lot more spread than when the Poisson regression is used. Applying a Poisson instead of a negative binomial regression when the *Y*-data is NB-distributed has a similar effect.

From the bottom two panels in Fig. 1 it can be learned that, not surprisingly, for both types of regression, the distribution of estimated values of $\beta$ when the *Y*-data is NB-distributed is a lot wider than when the *Y*-data is Poisson-distributed (note the different scale of the vertical axis for the top and bottom rows). We can then conclude that appropriate knowledge of the distribution of *Y* can help us obtain more precise estimates for the parameters of the scaling law between *X* and *Y*.

### Samples where not all values of *Y* satisfy the same scaling law

**Figure 2:**
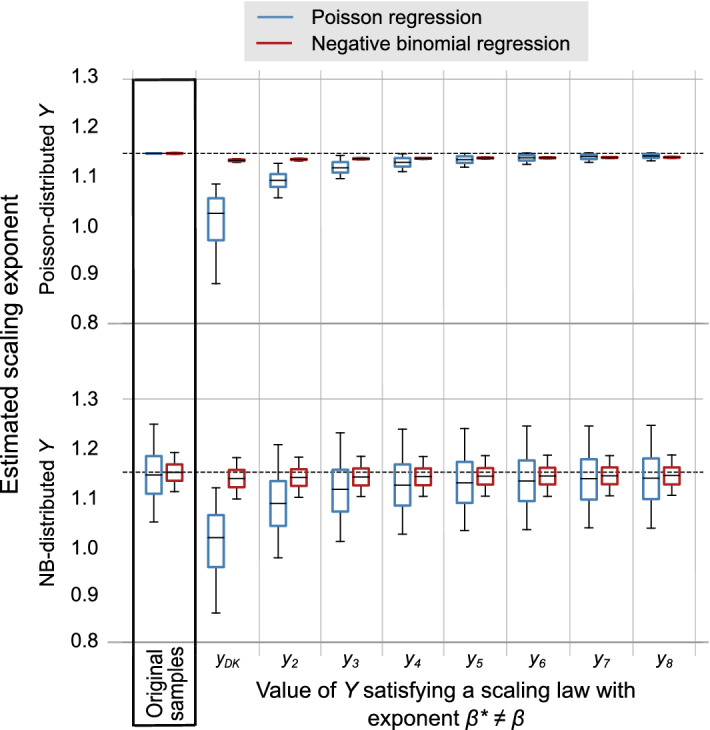
Boxplots showing the distribution of estimated values for parameter $\beta$ from each of the $m=1000$ samples with $n=500$. The *X*-data in the samples contains a dragon-king. The horizontal axis represents the value of *Y* that has been generated according to either a Poisson or a NB distribution with $E[Y|X] = \alpha X ^{\beta ^*}$, where $\alpha = 0.01$ and $\beta ^*=0.5$. The rest of values of *Y* are generated according to a Poisson or a NB distribution with $E[Y|X] = \alpha X ^{\beta }$, where $\beta = 1.15$. The vertical axis represents the values of the estimated scaling exponents. The bottom and top of the box indicate the first and third quartiles, the middle line in the box is the median and the upper and lower limits correspond to the 5th and 95th percentiles, giving a 90% confidence interval. The horizontal dashed line represents the true value of $\beta$, used to generate the synthetic data samples.

Figure 1 shows that, as long as *X* and *Y* satisfy the same scaling law for all the cities in each sample, the presence of dragon-kings does not have an impact in the estimated scaling exponent, regardless of the type of regression used for the estimation. In Fig. 2 we explore the situation where the value of *Y* corresponding to a specific city in each sample satisfies a scaling law with exponent $\beta ^*=0.5$ instead of $\beta =1.15$, as the rest of cities in the sample. The boxplots are obtained by estimating the scaling exponent from $m=1000$ samples. The first pair of boxplots on both the top and bottom panels, highlighted, correspond to samples with one dragon-king where all the values of *Y* satisfy Eq. (2). The rest of boxplots correspond to samples where $y_{DK}$ or $y_i$ for $i>2$ have been generated so that they satisfy $E[Y|X] = \alpha X ^{\beta ^*}$, with $\beta ^* = 0.5$.

We observe the largest discrepancy with the true parameters for the case where the dragon-king does not satisfy Eq. (2). The Poisson regression is particularly sensitive to the effect of the dragon-king. This is especially the case when *Y* is Poisson-distributed. The negative binomial regression is not as sensitive to the effect of the dragon-king, giving estimated values of the scaling exponent which are much closer to the true one. Generally, we observe that if a city with large population size deviates from the scaling law followed by the rest of cities, the effect on the estimated scaling exponents is more significant. The larger the city’s population size, the more this tendency becomes apparent.

## Discussion

From the results presented above, we conclude that the presence of dragon-kings in the *X* variable does not affect the value of the estimated exponent $\beta$ as long as *Y* satisfies the same scaling law for all the cities under consideration.

However, dragon-kings tend to display a different behaviour from other cities so, in real scenarios, *Y* might not necessarily follow the same scaling law as the rest of cities. If this happens, the estimated values of the scaling parameters given by the Poisson regression will be further from the true values than those given by the NB regression. This is due to the fact that the former regression method gives more importance to the largest data points. The Poisson or negative binomial regressions involve maximising a likelihood function via the IWLS method. This, in turn, involves successive iterations of a weighted least squares problem^24^. The weights assigned to different data points dictate their relative importance in determining the estimated parameters at each iteration of the IWLS method. Verhoef and Boveng^24^ show that the weight corresponding to the city with population $x_i$ in the *j*th iteration is $w_i^{j} = \mu _i^{j}/(1 + k^{j}\mu _i^{j})$. For the Poisson regression, this weight is $w_i^{j} = \mu _i^{j}$. The weights in the Poisson regression increase in proportion to $\mu _i^{j}$, while the weights in the negative binomial regression tend to $1/k^{j}$ for larger values of $\mu _i^{j}$. Cities with larger values of *X*, such as the dragon-kings, have corresponding larger expected values of *Y* and will therefore receive relatively more weight in the Poisson regression.

The findings of this paper raise the following dilemma. Regression methods that place relatively less weight on the larger city population sizes, such as the negative binomial regression, will produce estimates of the scaling parameters which are closer to the true values. But, when it comes to cities, the interest is often in the really large fast-growing cities that host a significant percentage of a country’s population. If the tendency is for large cities to evolve towards the dragon-king status, then scaling laws are not a good model, as there is an inherent flaw in the parameter estimation procedure.

In conclusion, if estimation techniques that give more weight to the larger entities are used, then the estimated exponents may be invalid unless the same scaling law is satisfied by all the cities in the urban system. But, if an estimation technique that does not give as much weight to these ‘outlier’ cities which are prone to deviate from the mainstream behaviour, then the most interesting part of the scaling law risks being neglected.
